# The efficacy and safety of yukmijihwang-hwan (Liuweidihuang-wan) for type 2 diabetes mellitus without complications: A protocol for systematic review and meta-analysis

**DOI:** 10.1097/MD.0000000000029087

**Published:** 2022-03-18

**Authors:** Jisoo Baek, Jinmi Kim, Seonmi Shin, Chungsik Cho

**Affiliations:** ^a^ *Department of Internal Korean Medicine, College of Korean Medicine, Dae-Jeon University, Daejeon, Republic of Korea,* ^b^ *Department. of Internal Korean Medicine, College of Korean Medicine, Se-Myung University, Chungcheongbukdo, South Korea.*

**Keywords:** antidiabetic effect, liuweidihuang-wan, meta-analysis, protocol, systematic review, type 2 diabetes mellitus, yukmijihwang-hwan

## Abstract

**Background:**

This systematic review and meta-analysis of randomized controlled trials (RCTs) will aim to assess the efficacy and safety of Yukmijihwang-hwan for type 2 diabetes without complications.

**Methods:**

To identify eligible studies, we will perform a systematic search of the following electronic databases: MEDLINE (PubMed), EMBASE, the Cochrane Central Register of Controlled Trials, China National Knowledge Infrastructure, Citation Information by NII, Korean Information Service System, Korean Medical Database, Oriental Medicine Advanced Searching Integrated System, and ScienceON. Search terms will include “Type 2 Diabetes” for participants as well as “Yukmijihwang-tang” or “Liuwei dihuang tang” for interventions. Two independent researchers will perform data extraction and assessment using Cochrane’s risk of bias tool, with disagreements being resolved through discussions with a third researcher.

**Results:**

This study will evaluate the antidiabetic effects of Yukmijihwang-hwan from 3 perspectives (blood glucose level, insulin resistance, and β-cell function) as well as its safety by reviewing the reported adverse effects.

**Conclusion:**

This systematic review will provide evidence regarding the antidiabetic efficacy and safety of Yukmijihwang-tang in type 2 diabetes mellitus.

## 1. Introduction

Type 2 diabetes mellitus (T2DM) is a chronic, progressive metabolic disease that is characterized by the presence of hyperglycemia, insulin resistance, and decreased β-cell function. Generally, most patients with T2DM are asymptomatic; however, its clinical symptoms include polyuria, polyphagia, polydipsia, and weight loss. The ultimate treatment goal for T2DM is the prevention of complications, including nephropathy, neuropathy, and diabetic foot. Conventional treatment strategies for T2DM include patient education, self-blood glucose measurement, lifestyle modifications such as dietary and exercise therapies, oral medication, and insulin injection.^[1,2]^

There has been an annual increase in the prevalence of diabetes. In 2019, approximately 463.0 million adults aged 20 to 79years worldwide (9.3% of all adults in this age group) have diabetes. Based on the current trend, the global diabetes prevalence is projected to be 10.9% in 2045. Additionally, the diabetes-related health expenditure increased from $232 billion in 2007 to $760 billion in 2019. The International Diabetes Federation predicts that the medical expenditure will increase to $825 billion by 2030 and $845 billion by 2045.^[3]^ Taken together, there has been a gradual increase in the prevalence and medical cost of diabetes. Thus, T2DM can be a social and economic burden, and there has been increasing interest in methods for improving hyperglycemia in T2DM, with several studies being conducted on East Asian Traditional Medicine (EATM).

From the perspective of EATM, T2DM is included in the category of “So-gal”; moreover, the main cause of “So-gal” is “kidney deficiency.” Yukmijihwang-hwan (YM) is widely used to treat diseases caused by kidney deficiency and is the main drug used for T2DM treatment.^[4]^

There has been increasing evidence regarding the efficacy of YM for T2DM. YM extract administration to diabetic rats showed an anti-diabetic effect through inhibition of a-amylase and a-glucosidase activity.^[5]^ Additionally, YM administration to rats with T2DM exerted protective effects on beta cells by suppressing NF-κB expression in pancreatic islets.^[6]^ Additionally, YM administration was found to improve T2DM-related insulin resistance by increasing IRS-2 expression in the insulin signaling pathway or regulating the PI3K/Akt signaling path-way.^[7,8]^

Systematic reviews have found that YM has significant effects on patients with T2DM,^[9-14]^ however, they only focused on improvements in blood glucose levels rather than insulin resistance and beta-cell dysfunction. Therefore, we will conduct a systematic review and meta-analysis to verify the safety and efficacy of YM for hyperglycemia, insulin resistance, and β-cell function in patients with T2DM without complications.

## 2. Materials and methods

### 2.1. Study registration

This protocol is registered in the Open Science Framework (OSF), with the registration doi. The protocol will be reported according to the Preferred Reporting Items for Systematic Reviews and Meta-Analyses guidelines.

### 2.2. Inclusion/exclusion criteria

We will compose the clinical question following the Population, Intervention, Comparison, and Outcomes-SD form to conduct a systematic review following the guidelines of the National Evidence-based Healthcare Collaborating Agency (NECA).^[15]^

#### 2.2.1. Type of studies.

We will only include randomized controlled trials (RCTs) regarding T2DM treatment using YM, regardless of the language or publication restriction. We will exclude other studies such as case reports, case series, nonhuman studies, nonRCTs, protocols, pilot studies, uncontrolled trials, and reviews.

#### 2.2.2. Type of participants.

We will include patients with clinical symptoms of T2DM, including polydipsia, polyuria, and weight loss, or patients with T2DM according to the 1999 WHO criteria or ADA standard. There will be no limitations regarding age, disease duration, disease severity, and sex. We will exclude patients with diabetes-related complications, including neuropathy, nephropathy, retinopathy, and obesity; patients diagnosed with gestational diabetes; and patients diagnosed with type 1 diabetes mellitus.

#### 2.2.3. Types of interventions

##### 2.2.3.1. Experimental intervention.

We will include studies where the experimental intervention is YM as monotherapy with or without conventional treatment, including oral antidiabetic agents, insulin injection, and lifestyle improvement. Any YM composition or fusion will be allowed. We will exclude studies using YM as an adjuvant treatment to other herbal medicines, as well as those involving acupuncture, acupressure, catgut embedding, moxibustion, and Qigong therapy as experimental adjuvant treatments.

##### 2.2.3.2. Control intervention.

We will include studies where the control intervention is conventional treatment, including oral antidiabetic agents, insulin injections, and lifestyle improvement.

#### 2.2.4. Types of outcome measurements

##### 2.2.4.1. Primary outcomes.

The primary outcome measures will include fasting plasma glucose, 2-hour postprandial glucose, and glycated hemoglobin A1c.

##### 2.2.4.2. Secondary outcomes.

The secondary outcome measurements will include fasting insulin, homeostasis model assessment for insulin resistance, homeostasis model assessment for beta-cell function, and the number of adverse effects.

### 2.3. Searching methods for included studies

#### 2.3.1. Electronic searches.

We will search the following databases from their inception until August 31, 2021: the Cochrane Central Register of Controlled Trials, China National Knowledge Infrastructure, Citation Information by NII, EMBASE, Oriental Medicine Advanced Searching Integrated System, Korean Medical Database, Korean Studies Information Service System, Medline via PubMed, ScienceON, and RISS. The search terms included “type 2 diabetes mellitus” for participants; “yukmijihwang-tang,” “liuwei dihuang-tang,” or “rokumijiogan” for interventions; and “randomized clinical trial” for study design. (Tables 1-3)

**Table 1:** Search strategy for the PubMed.

**No.**	**Search items**
#1	“Diabetes Mellitus”[MeSH Terms]
#2	“Diabetes *”[Title/Abstract]
#3	“Diabetes”[Title/Abstract] OR “T2DM*”[Title/Abstract] OR “NIDDM”[Title/Abstract] OR “Type 2”[Title/Abstract] OR “Type II” [Title/Abstract]
#4	“non insulin* depend*”[Title/Abstract] OR “non-insulin* depend*”[Title/Abstract] OR “non insulin?depend*”[Title/Abstract] OR “non-insulin?depend*”[Title/Abstract]
#5	#1 OR #2 OR #3 OR #4
#6	“Yukmijihwang-tang” [Title/Abstract] OR “Yukmijiwhang-tang” [Title/Abstract] OR “Yukmi” [Title/Abstract] OR “Yukmijihwang-hwan[Title/Abstract]
#7	“Liuwei Dihuang“[Title/Abstract]
#8	“Rokumi-gan”[Title/Abstract] OR “Rokumi-jio-gan” [Title/Abstract]
#9	#6 OR #7 OR #8
#10	#5 AND #9

**Table 2:** Search strategy for the EMBASE.

**No.**	**Search items**
#1	diabetes AND mellitus
#2	diabet$:ab, ti
#3	(‘non insulin* depend*’ or ‘non insulin?depend*’):ab,ti
#4	(IDDM or NIDDM or MODY or T2DM or T2D). ab,ti
#5	#1 OR #2 OR #3 OR #4
#6	(Yukmijihwang-tang OR Yukmijiwhang-tang OR Yukmi OR Yukmijihwang-hwan):ab, ti
#7	‘Liuwei Dihuang’:ab, ti
#8	(Rokumi-gan OR Rokumi-jio-gan):ab, ti
#9	#6 OR #7 OR #8
#10	#5 AND #9

**Table 3:** Search strategy for the CENTRAL.

**No.**	**Search items**
#1	MeSH descriptor: [Diabetes Mellitus] explode all trees
#2	(diabet*)
#3	(IDDM or NIDDM or MODY or T2DM or T2D)
#4	(non insulin* depend* or non insulin depend* or non insulin?depend* or non insulin?depend)
#5	(insulin* depend* or insulin?depend*)
#6	#1 OR #2 OR #3 OR #4 OR#5
#7	(Yukmijihwang-tang OR Yukmijiwhang-tang OR Yukmi OR Yukmijihwang-hwan):ti, ab, kw
#8	Liuwei Dihuang:ti, ab, kw
#9	(Rokumi-gan OR Rokumi-jio-gan):ti, ab, kw
#10	#7 OR #8 OR #9
#11	#6 AND #10

#### 2.3.2. Searching other resources.

We will review the reference lists of the included studies to identify additional studies.

### 2.4. Data collection and analysis

#### 2.4.1. Literature selection.

All collected studies will be imported into the literature management software Endnote 20.0.1 software (Clarivate Analytics, Boston, MA). After removing duplicate studies, 2 independent researchers will screen the titles and/or abstracts to identify studies that meet the inclusion criteria. Subsequently, both researchers will perform full-text reviews of the studies retrieved from the first screening. Disagreements will be resolved by consulting a third researcher. Figure 1 shows the selection process for eligible studies.

**Figure:**
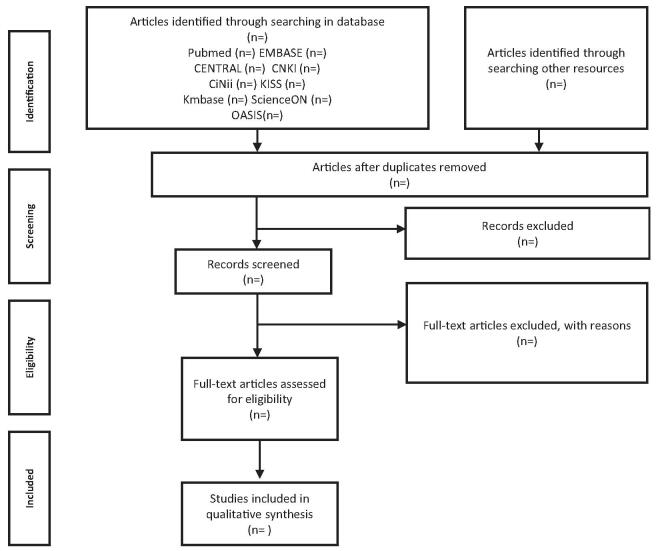
**Figure 1.** PRISMA Flow Chart of Selection Process.

#### 2.4.2. Data extraction.

After retrieving eligible studies, we will extract the following data using a pre-specified data extraction form: first author, publication year, study design, intervention, comparison, duration, follow-up, outcome measures, results, and adverse events. The extracted information will be summarized and organized using Microsoft Excel 2020. In case of missing data, the original authors will be contacted.

#### 2.4.3. Risk of bias assessment.

Two independent reviewers will evaluate the quality of the included studies using Cochrane’s Risk of Bias (RoB) tool following the NECA guidelines.^[15]^ To reduce differences in opinion resulting from interpretation, we will use an RoB tool translated into Korean. Disagreements will be resolved by consulting a third researcher. The RoB tool evaluates the following biases:

1. Random sequence generation
2. Allocation concealments
3. Participants’ blinding.
4. Blinding personnel.
5. Blinding of outcome assessment
6. Incomplete outcome data
7. Selective reports
8. Other bias

#### 2.4.4. Measurement of intervention effect.

Continuous data will be presented as the mean difference or standardized mean difference with a 95% confidence interval (CI). Dichotomous data will be presented as the risk ratio with a 95% CI.

#### 2.4.5. Subgroup analysis.

In case sufficient data are available, we will perform subgroup analysis based on the sole use of YM, oral antidiabetic agents, and insulin injections.

#### 2.4.6. Heterogeneity measurement.

Among-study heterogeneity will be evaluated using the Chi-Squared test and *I*^2^ statistic. We will grade the heterogeneity as follows:

1. *I*^2^ < 25%: low grade
2. 25% ≤ *I*^2^ ≤ 50%: moderate grade
3. *I*^2^ ≥ 75%: high grade

#### 2.4.7. Data synthesis.

We will perform a meta-analysis to draw significant conclusions regarding the clinical effect of YM. In case of homogeneity in the study design, it is possible to synthesize the data regarding the intervention group, control group, and outcome measurement. Specifically, the fixed-effects model will be used when there are ≤ 4 included studies or in case of nonsignificant heterogeneity. Contrastingly, the random-effects model will be used for meta-analysis in case of significant heterogeneity (*I*^2^ > 50%).

We will perform the meta-analysis using RevMan version 5.4 (Copenhagen, The Nordic Cochrane Center, the Cochrane Collaboration, 2020).

### 2.5. Ethics and dissemination

No ethical approval is required for this protocol since we will not use individual patient data. The results will be published in a peerreviewed journal and presented at a relevant conference.

## 3. Discussion

Recent systematic reviews have suggested that YM has antidiabetic effects in patients with T2DM.^[9-14]^ However, they only confirmed the effect of YM on patients taking metformin with other antidiabetic agents.^[12-14]^ Therefore, the effect of YM alone or with insulin treatment remains unclear. Additionally, it remains unclear whether T2DM improves insulin resistance and beta-cell function. Therefore, the proposed study will present updated evidence regarding the efficacy and safety of YM for T2DM without complications in terms of regulating glucose levels and improving insulin resistance and beta-cell function.

This study has several potential limitations. First, the quality of RCTs may be low due to performance bias since we will include studies with a nonplacebo control group. Second, since the applied YM formulation was not standardized across the studies, there may be high among-study heterogeneity. Nonetheless, this study may provide evidence for recommending YM to patients with T2DM without complications. Additionally, this study could provide future research directions.

## Acknowledgments

The authors would like to thank Editage (www.editage.co.kr) for English language editing.

## Author contributions

**Conceptualization:** Seon-Mi Shin, Chung-Sik Cho.

**Data curation:** Jisoo Baek, Jin-Mi Kim, Chung-Sik Cho.

**Formal analysis:** Jisoo Baek, Chung-Sik Cho.

**Funding acquisition:** Seon-Mi Shin, Chung-Sik Cho.

**Writing** - **original draft:** Jisoo Baek, Chung-Sik Cho.

**Writing** - **review & editing:** Jisoo Baek, Jin-Mi Kim, Seon-Mi Shin, Chung-Sik Cho.
